# Circumscribed Meningeal Melanocytoma of the Craniovertebral Junction: A Case Report and Focused Literature Review

**DOI:** 10.3390/reports9040323

**Published:** 2026-09-24

**Authors:** Maciej Laskowski, Piotr Paździora, Łukasz Antkowiak, Jan Herzyk, Anna Zioła-Paździora, Maciej Wojtacha

**Affiliations:** 1 Department of Neurosurgery, Saint Barbara Regional Hospital and Trauma Center in Sosnowiec, Medical University of Silesia, 41-214 Sosnowiec, Poland; 2 Department of Neurosurgery, University Clinical Center, Faculty of Medical Sciences in Katowice, Medical University of Silesia, 40-752 Katowice, Poland; 3 Health Promotion and Obesity Management Unit, Department of Patophysiology, Faculty of Medical Sciences in Katowice, Medical University of Silesia, 40-752 Katowice, Poland; 4 Department of Pediatric Surgery, Upersiliesian Children’s Health Center in Katowice, 40-752 Katowice, Poland

**Keywords:** meningeal melanocytoma, melanocytic tumor, craniovertebral junction

## Abstract

**Background and Clinical Significance:** Meningeal melanocytoma is an exceptionally rare, typically benign melanocytic tumor of the central nervous system. Its clinical management becomes particularly challenging when located at the craniovertebral junction, where proximity to critical neurovascular structures limits the extent of safe surgical resection. Due to its rarity and complex anatomical location, each case contributes valuable insight into optimal diagnostic and therapeutic strategies. We report a case of circumscribed meningeal melanocytoma arising at the craniovertebral junction and provide a focused review of previously reported cases, with particular emphasis on clinical presentation, radiological characteristics, surgical challenges, treatment strategies, and outcomes; **Case Presentation:** A 65-year-old Caucasian woman presented with progressive binocular diplopia. Magnetic resonance imaging revealed two contrast-enhancing extra-axial lesions at the level of the foramen magnum, with one dominant mass. The patient underwent near-total microsurgical resection of the dominant lesion under intraoperative neurophysiological monitoring. Histopathological and immunohistochemical evaluation confirmed the diagnosis of meningeal melanocytoma. In the postoperative period, the patient developed transient lower cranial nerve dysfunction, which improved with conservative treatment. Follow-up imaging demonstrated a stable residual tumor. Given the subtotal nature of resection and the anatomically complex location, adjuvant proton beam radiotherapy was recommended; **Conclusions:** Meningeal melanocytoma of the craniovertebral junction poses distinctive diagnostic and surgical challenges because of its proximity to critical neurovascular structures. The available literature supports maximal safe resection as the primary treatment strategy, with adjuvant radiotherapy considered in selected patients with residual disease. Long-term radiological surveillance is warranted because late recurrence and malignant transformation have been reported.

## 1. Introduction and Clinical Significance

Meningeal melanocytoma is a rare primary tumor of the central nervous system arising from leptomeningeal melanocytes derived from neural crest cells [1]. These cells are predominantly distributed along the ventrolateral surface of the medulla oblongata and upper spinal cord, which explains the predilection of these tumors for the posterior cranial fossa and cervical spine [2]. According to the World Health Organization (WHO) classification, meningeal melanocytoma is classified as a grade I melanocytic tumor with low malignant potential [3]. According to the 2021 World Health Organization (WHO) Classification of Tumors of the Central Nervous System, primary meningeal melanocytic tumors are divided into diffuse meningeal melanocytic neoplasms and circumscribed meningeal melanocytic neoplasms, with the latter including meningeal melanocytoma and meningeal melanoma [3]. Circumscribed meningeal melanocytic tumors are characteristically associated with alterations involving GNAQ, GNA11, PLCB4, or CYSLTR2 [3].

Rare multifocal cases have also been described [4]. The estimated incidence of meningeal melanocytoma is approximately one case per 10 million individuals, accounting for about 0.06–0.1% of all brain tumors [5]. The posterior fossa and upper cervical spinal cord are the most frequently reported locations, whereas involvement of the craniovertebral junction is rare [5].

Given the rarity of meningeal melanocytoma at the craniovertebral junction and the limited literature specifically addressing this location, the aim of this report was to present a surgically challenging case of circumscribed meningeal melanocytoma of the craniovertebral junction and to contextualize it through a focused review of previously reported cases. Particular emphasis was placed on clinical presentation, MRI characteristics and differential diagnosis, surgical challenges, molecular features, treatment strategies, and clinical outcomes.

## 2. Case Presentation

A 65-year-old left-handed Caucasian woman, a retired office worker, with a medical history of arterial hypertension, dyslipidemia, and thyroid nodules, was referred for a routine ophthalmological examination prior to the prescription of new corrective glasses. Apart from mild age-related refractive errors, the ophthalmological examination revealed no abnormalities, except that the patient reported diplopia when looking into the distance to the left. She was subsequently referred to the neurosurgical outpatient clinic.

On neurological examination, she was alert and fully oriented, with coherent and logical speech. Motor function was normal, movements were smooth and coordinated, she maintained a stable stance during the Romberg test, and the finger-to-nose test was normal. No signs of cranial nerve dysfunction were detected.

Her past medical history was notable for Achilles tendon surgery performed 20 years earlier. The family and social histories were unremarkable. Her regular medications included a fixed-dose combination of rosuvastatin/ezetimibe (40/10 mg) and telmisartan 40 mg daily. Because of her visual symptoms, magnetic resonance imaging (MRI) of the brain was performed.

Magnetic resonance imaging revealed a contrast-enhancing extra-axial lesion measuring 17 × 15 × 27 mm extending from the foramen magnum toward the fourth ventricle, causing left-sided compression of the medulla oblongata (Figure 1).

A second lesion of similar morphology, approximately 6 mm in diameter, was identified on the ventral aspect of the spinal canal at the level of the odontoid process (Figure 2).

Differential diagnoses included meningioma, melanocytoma, and melanotic schwannoma.

Given the tumor’s proximity to the brainstem and lower cranial nerves, stereotactic radiosurgery was considered unsafe. A multidisciplinary team recommended primary microsurgical resection.

The patient was positioned prone, with the head secured in a Mayfield three-pin fixation system, slightly rotated to the left and flexed toward the chest. Following standard skin preparation and sterile draping, a midline linear skin incision was made from the inion to the C2 spinous process. The paraspinal muscles were dissected using a monopolar device, exposing the left posterior cranial fossa and the posterior arch of C1.

A left-sided suboccipital craniectomy was performed using two burr holes, which were subsequently enlarged with a Kerrison rongeur. The left portion of the posterior C1 arch was then removed to approximately half of its width, providing additional surgical exposure. The position of the vertebral artery was identified using intraoperative Doppler ultrasonography. The operating microscope was subsequently introduced.

The dura mater was opened within the limits of the bony exposure and reflected. The lesion appeared macroscopically similar to a meningioma. Intraoperative neurophysiological monitoring (IOMN) was used throughout the procedure, with responses obtained from cranial nerves XI and XII. Tumor resection was performed progressively, with initial debulking and central tumor evacuation followed by microsurgical dissection from the surrounding neurovascular structures. In areas where strong neurophysiological responses from cranial nerves XI and XII were obtained, tumor manipulation was minimized to reduce the risk of neurological injury.

At the superior pole of the lesion, limited cerebellar retraction was required to obtain adequate visualization. The vertebral artery was identified, and the tumor was meticulously separated from the vascular structures and brainstem by arachnoid dissection using microsurgical instruments, with both sharp and blunt techniques employed as appropriate. Several episodes of intraoperative bradycardia occurred during tumor manipulation. Given the strong neurophysiological responses from cranial nerves XI and XII and the close adherence of the tumor to these structures, further resection in these areas was considered unsafe and was therefore discontinued. A near-total resection was consequently achieved.

After meticulous hemostasis was obtained, dural reconstruction was performed using TachoSil and DuraGen. The muscular, subcutaneous, and skin layers were closed in anatomical layers, followed by application of a sterile dressing.

Postoperatively, the patient developed mild dysphagia and dysphonia. Flexible laryngoscopy revealed left vocal fold paralysis without aspiration. Symptoms gradually improved with voice therapy. Histopathological examination demonstrated nests of melanocytic cells with low mitotic activity and minimal atypia. Immunohistochemistry showed positivity for Melan A, HMB45, SOX10, and S100, with a low Ki-67 index (<2%), confirming meningeal melanocytoma.

At 6-month follow-up, imaging demonstrated stable residual tumor adjacent to the vertebral artery and ventral brainstem, with no progression of the second lesion (Figure 3 and Figure 4). Adjuvant proton beam radiotherapy was recommended. The lesion was not subjected to microscopic examination because no material was obtained for histopathological evaluation.

Following subtotal tumor resection, the patient was referred for postoperative radiotherapy and, following multidisciplinary evaluation, was qualified for proton beam radiotherapy. Treatment was initiated approximately 8 months after surgery.

The residual tumor in the left cerebellopontine angle was treated to a total dose of 54 Gy RBE in 30 fractions (1.8 Gy RBE per fraction). In addition, the meningeal lesion located within the spinal canal at the right C1/C2 level was treated to 50.4 Gy RBE in 28 fractions (1.8 Gy RBE per fraction). The treatment volumes were delineated based on the postoperative imaging findings and encompassed the radiologically identified residual tumor in the left cerebellopontine angle and the meningeal lesion at the right C1/C2 level.

The treatment was well tolerated, with no significant acute treatment-related toxicity. A grade 1 skin reaction was observed in the irradiated area. The patient completed the planned proton beam radiotherapy and was discharged in good general condition.

Although diplopia was the presenting symptom, no dysfunction of cranial nerves III, IV, and VI was identified clinically or radiologically. Therefore, a direct causal relationship between the tumor and diplopia could not be definitively established.

## 3. Discussion

Meningeal melanocytoma is a rare primary melanocytic neoplasm of the central nervous system with a predilection for the posterior fossa and upper cervical spinal canal. Although histologically well-differentiated tumors usually demonstrate indolent behavior, their clinical course is not uniformly benign, as local recurrence and, less frequently, malignant transformation have been reported. Consequently, both the anatomical location of the tumor and the extent of surgical resection are important determinants of long-term management [6,7].

Meningeal melanocytomas involving the craniovertebral junction (CVJ) represent a particularly uncommon subgroup. To contextualize the present case, we performed a focused literature search for previously reported meningeal melanocytomas involving this anatomical region. Relevant reports were identified through PubMed/MEDLINE and reference-list screening, and their clinical, radiological, surgical, and outcome characteristics are summarized in Table 1. Clinical presentation was heterogeneous and largely reflected compression of the medulla oblongata and upper cervical spinal cord. Headache, gait disturbance, sensory abnormalities, and limb weakness were among the most frequently reported manifestations, whereas progressive myelopathy or quadriparesis occurred in more advanced cases [5,8,9]. The nonspecific nature of these symptoms may complicate preoperative recognition of this rare entity.

The MRI appearance of meningeal melanocytoma is closely related to its melanin content. Most reported lesions demonstrate the characteristic combination of T1-weighted hyperintensity and T2-weighted hypointensity, although substantial variability may occur [8,10]. Following gadolinium administration, these tumors generally demonstrate marked enhancement [10]. Nevertheless, MRI findings are not specific, and the differential diagnosis at the CVJ includes meningioma, melanotic schwannoma, and malignant melanoma [8,9,10]. Consequently, definitive diagnosis requires histopathological and immunohistochemical evaluation [10].

Gross-total resection was achieved in the majority of previously reported CVJ cases [5]. However, surgical feasibility is strongly influenced by the exact anatomical relationship of the lesion to the medulla, upper cervical spinal cord, lower cranial nerves, and vertebral vascular structures [5,9]. The present case was particularly challenging because the dominant lesion was closely related to the lower cranial nerves and adjacent vascular structures, while an additional radiologically similar lesion was located ventrally at the C1/C2 level. Intraoperative neurophysiological responses from cranial nerves XI and XII and close tumor adherence to critical neurovascular structures prompted intentional preservation of a small residual component. Although most previously reported patients remained recurrence-free during the available follow-up, late recurrence and malignant transformation have also been documented, emphasizing the need for prolonged radiological surveillance [7,8].

**Table 1:** Summary of the previously reported cases of meningeal melanocytoma involving the craniovertebral junction.

Author, Year	Age, Sex	Location	Clinical Presentation	MRI Features	EOR	Adjuvant RT	Follow-Up Duration and Outcome
Limas and Tio, 1972 [11]	71/M	Foramen magnum–C1	Headache; left lower-extremity weakness	NR	Autopsy diagnosis	NR	NR; died
Naul et al., 1991 [12]	68/F	Foramen magnum	Suboccipital headache, dizziness, nausea/vomiting, gait difficulty; quadriparesis	T1 isointense; T2 hypointense; homogeneous enhancement	GTR	No	10 m; no recurrence
Uematsu et al., 1992 [13]	62/M	Foramen magnum–C1	Gait disturbance	T1 hyperintense; T2 hypointense	GTR	No	18 m; no recurrence
O’Brien et al., 1995 [14]	49/M	Foramen magnum–C3	Poor vision	NR	GTR	No	Recurrence at 8 y; died at 9 y
Hirose et al., 1997 [15]	66/M	Foramen magnum–C1	Gait disturbance/ataxia; bilateral hand/upper-extremity numbness	T1 isointense; T2 hypointense; enhancement	GTR	No	8 m; no MM recurrence; died of unrelated renal cell carcinoma
Châabane et al., 2003 [16]	48/M	Foramen magnum–C1/C2	Bilateral upper-extremity paresthesias in C4–C5 distribution, progressive for 6 months; quadripyramidal syndrome	T1 hyperintense; T2 markedly hypointense; moderate contrast enhancement	NR	NR	NR
Offiah et al., 2006 [17]	25/F	Cisterna magna/posterior foramen magnum	Headache, nausea, vomiting	T1 hyperintense; T2 hypointense	GTR	No	6 m; no recurrence
Fan et al., 2012 [18]	48/M	Foramen magnum	Headache; left-sided weakness and numbness	T1 hyperintense; T2 isointense	GTR	No	12 m; no recurrence
Bhargava et al., 2013 [9]	55/M	CVJ, extending toward C2–C3 neural foramen	Progressive quadriparesis	T1 hyperintense; T2 hypointense	NTR	No	10 m; no reported progression; residual left upper-extremity weakness
Sodhi et al., 2014 [19]	22/M	Ventral foramen magnum, dural attachment to C2	Headache; intermittent diplopia; right neck tilt	Predominantly T1/T2 isointense with intense enhancement; focal T1-hyper/T2-hypointense nodule; vertebral arteries involved	GTR	45 Gy	6 m; no recurrence
Yang et al., 2016—case 1 [20]	51/M	Foramen magnum–C2	Right limb weakness; four-limb numbness	T1 hyperintense; T2 hypointense	GTR	No	25 m; no recurrence
Yang et al., 2016—case 2 [20]	26/M	Foramen magnum–C1	Vomiting; bilateral upper-extremity numbness	T1 hyperintense; T2 hypointense	GTR	No	31 m; no recurrence
Yang et al., 2016—case 3 [20]	39/M	Foramen magnum–C1	Four-limb weakness	T1 hyperintense; T2 hypointense	GTR	No	47 m; no recurrence
Lee et al., 2017 [21]	45/M	C1, intradural	Progressive neck pain → leg weakness, gait disturbance and hemiparesthesia	T1 hyperintense; T2 hypointense; contrast enhancement	GTR	No	6 m; no recurrence
Uramaru et al., 2021 [5]	44/M	Dorsal CVJ/foramen magnum–C1	Occipital headache, nausea, vomiting	T1 hyperintense; T2 hypointense; little/no apparent Gd enhancement	GTR	No upfront RT; salvage WBRT after malignant transformation [22]	Malignant transformation and leptomeningeal dissemination at 45 m; died 9 m later [22]
Chaharyn et al., 2022 [23]	64/F	Cervicomedullary junction, left-sided exophytic intra-axial lesion	1-y gait difficulty; right-sided dysesthesias	Enhancing cervicomedullary mass; amelanotic	NR	NR	NR
Deng et al., 2022 [24]	19/F	C1–C2, dumbbell-shaped	Headache, progressive upper- and lower-limb weakness, dyspnea	T1 hyperintense; mixed T2 signal; strong homogeneous enhancement	GTR	No upfront RT; salvage systemic therapy/CSI after recurrence	Local recurrence at 6 m; intracranial dissemination and malignant transformation at 9 m; died
Kimchi et al., 2025 [25]	44/M	Intramedullary cervicomedullary junction	Suboccipital pain; impaired hand dexterity; gait imbalance	T1 hyperintense; solid component T2 isointense; cystic component; homogeneous enhancement	NTR	45 Gy/25 fractions	13 m; stable residual tumor, no progression
Present case, 2026	65/F	Dominant foramen magnum lesion + second ventral C1/C2 lesion at odontoid level	Progressive binocular diplopia	Two contrast-enhancing extra-axial lesions	NTR	Proton RT: 54 Gy RBE/30 fx to residual; 50.4 Gy RBE/28 fx to second lesion	6 m postoperatively; stable residual tumor and second lesion, no progression

Legend: CVJ, craniovertebral junction; GTR, gross-total resection; NTR, near-total resection; RT, radiotherapy; WBRT, whole-brain radiotherapy; Gd, gadolinium; MM, meningeal melanocytoma; NR, not reported; m, months; y, years; Gy, gray; RBE, relative biological effectiveness; fx, fractions. Follow-up outcome refers to the last available clinical or radiological follow-up reported in the original publication or subsequent follow-up report.

Surgical resection remains the mainstay of treatment. The largest available pooled analysis, including 201 reported patients, showed that complete resection was associated with the most favorable recurrence-free outcome, whereas patients undergoing partial resection had a greater risk of subsequent progression [7]. Nevertheless, at anatomically complex sites such as the CVJ, gross total resection should not be pursued at the expense of permanent neurological morbidity. In the present patient, intraoperative adherence of the tumor to the posterior inferior cerebellar artery and electrophysiological responses from cranial nerves XI and XII justified intentional preservation of a small residual component. The transient postoperative dysphagia, dysphonia, and vocal fold paresis observed despite this conservative strategy further illustrate the functional vulnerability of this region.

The role of postoperative radiotherapy is less firmly established because evidence is derived predominantly from retrospective series, pooled case reports, and small institutional experiences. Nevertheless, the available literature consistently suggests a benefit in local tumor control when radiotherapy is used after subtotal resection or for recurrent disease [6,7,26]. In the updated analysis by Rades et al., outcomes after incomplete resection were superior when surgery was followed by radiotherapy compared with subtotal resection alone, and higher radiation doses appeared to provide better local control [6,26]. These findings support consideration of adjuvant irradiation in patients with macroscopic residual tumor when further surgery would carry substantial neurological risk.

The optimal radiation technique, however, remains undefined. Highly conformal approaches may be particularly attractive for lesions of the CVJ because of the close proximity of the medulla, upper cervical spinal cord, cranial nerves, and vertebral arteries. In the present case, proton beam radiotherapy was recommended with the aim of achieving adequate dose coverage of the residual tumor while limiting irradiation of surrounding critical structures. Importantly, this recommendation is based primarily on anatomical and dosimetric considerations; there is currently insufficient disease-specific evidence to demonstrate oncological superiority of proton therapy over modern photon-based radiotherapy in meningeal melanocytoma. Similarly, stereotactic radiosurgery may be appropriate for selected small, well-demarcated residual or recurrent lesions, but its use must be individualized when lesions directly abut critical neural structures.

Prognosis in meningeal melanocytoma seems to depend on both treatment-related and biological factors. Among treatment-related factors, the extent of resection remains the most consistently reported prognostic factor. In the updated analysis by Rades et al., gross-total resection was associated with improved local control and overall survival compared with subtotal resection, while postoperative radiotherapy significantly improved local control after subtotal resection [26]. Histopathological features associated with more aggressive biological behavior include increased mitotic activity, necrosis, and CNS invasion, although their individual prognostic significance remains incompletely defined [27]. Molecular alterations, including SF3B1, EIF1AX, and BAP1 mutations and chromosomal abnormalities, may further contribute to risk stratification, but their prognostic relevance in meningeal melanocytoma requires additional validation [27]. Long-term surveillance remains essential because late local recurrence and malignant transformation have been documented even after apparently successful treatment. In the present patient, low mitotic activity, minimal atypia, and a Ki-67 index below 2% represent favorable histopathological features; however, the residual tumor and the presence of a second radiologically similar lesion justify prolonged serial MRI follow-up.

At the molecular level, circumscribed meningeal melanocytic tumors are characterized predominantly by mutually exclusive activating alterations involving the Gαq signaling pathway, most commonly mutations in GNAQ and GNA11 and, less frequently, PLCB4 or CYSLTR2 [3,27]. These molecular features resemble those observed in uveal melanoma and may support the diagnosis of a primary meningeal melanocytic tumor. Additional alterations involving SF3B1, EIF1AX, and BAP1, as well as chromosomal abnormalities such as monosomy 3 and complex copy-number changes, have been described and may contribute to biological heterogeneity and potentially more aggressive behavior [27]. In contrast, alterations commonly associated with cutaneous melanoma, including BRAF mutations, are uncommon in circumscribed primary meningeal melanocytic tumors, which may be diagnostically useful when distinguishing these lesions from metastatic melanoma [27]. Molecular profiling was not available in the present patient and therefore molecular characterization of the tumor beyond histopathological and immunohistochemical assessment was not possible. In addition, preoperative cerebrospinal fluid cytology was not performed; consequently, occult leptomeningeal dissemination could not be assessed. These factors represent limitations of the present case.

In summary, the present case illustrates that treatment of CVJ meningeal melanocytoma requires a balance between oncological radicality and preservation of neurological function. Gross total resection remains the preferred treatment when safely achievable, whereas intentional subtotal resection followed by consideration of adjuvant radiotherapy represents a reasonable strategy when the tumor is inseparable from critical neurovascular structures. The unusual anatomy and multifocal appearance observed in our patient further emphasize the importance of individualized treatment planning and prolonged radiological surveillance.

## 4. Conclusions

Meningeal melanocytoma of the craniovertebral junction is a rare entity characterized by considerable heterogeneity in clinical presentation, radiological appearance, surgical accessibility, and long-term behavior. The available literature indicates that maximal safe resection remains the cornerstone of treatment, although gross-total resection may not be safely achievable when the tumor is closely associated with the brainstem, lower cranial nerves, or vascular structures. In such cases, intentional incomplete resection with consideration of adjuvant radiotherapy represents a reasonable management strategy. Although most reported patients have experienced favorable short-term outcomes, late recurrence and malignant transformation have been documented, supporting prolonged radiological surveillance. Molecular profiling may provide additional diagnostic and prognostic information, but its clinical significance requires further investigation. The present case further illustrates the importance of balancing oncological control with preservation of neurological function in this anatomically complex region.

## Figures and Tables

**Figure 1:**
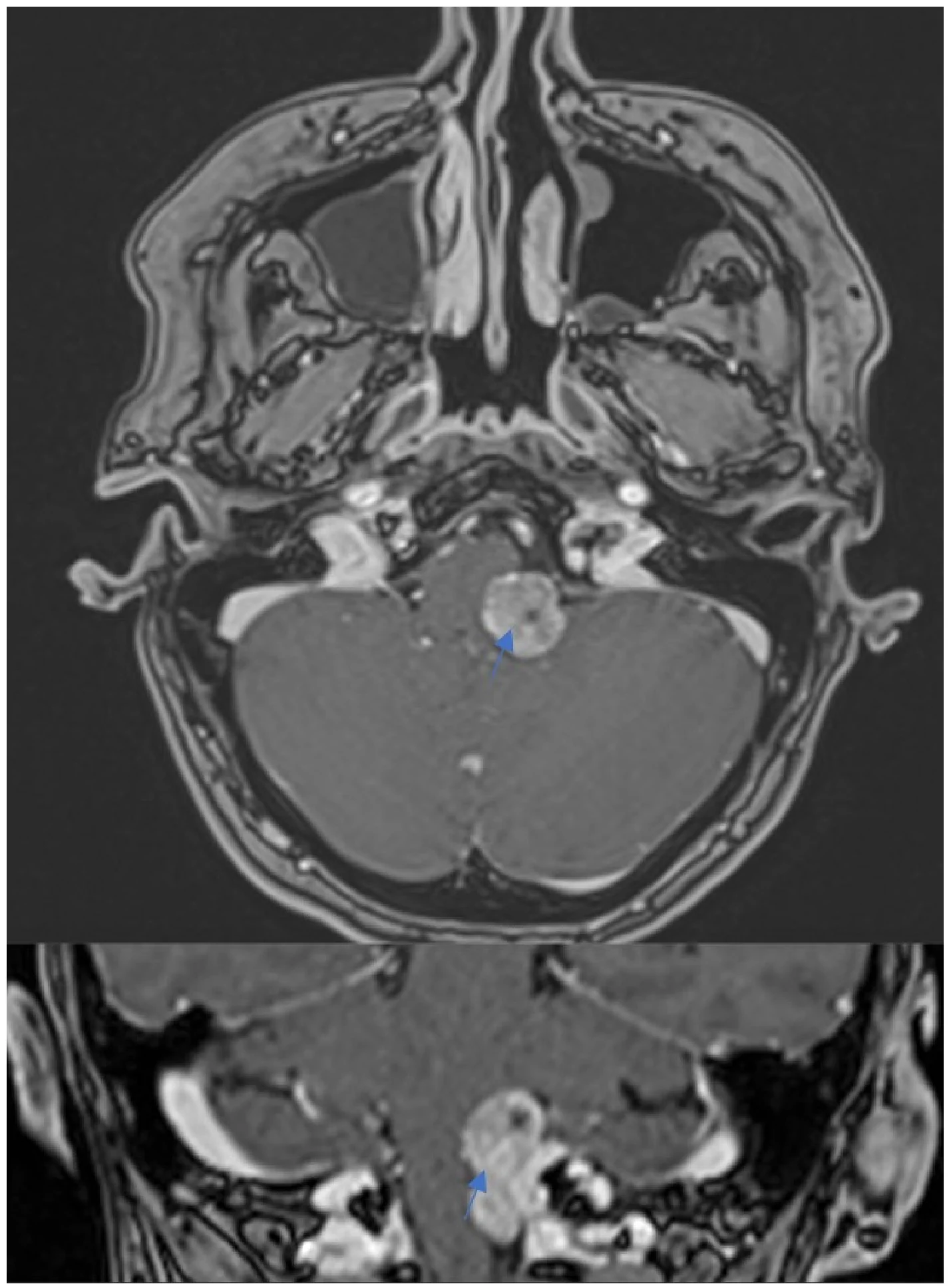
Preoperative post-gadolinium T1-weighted MRI showing an extra-axial lesion extending from the foramen magnum toward the fourth ventricle with compression of the medulla oblongata–axial and coronal plane (blue arrows).

**Figure 2:**
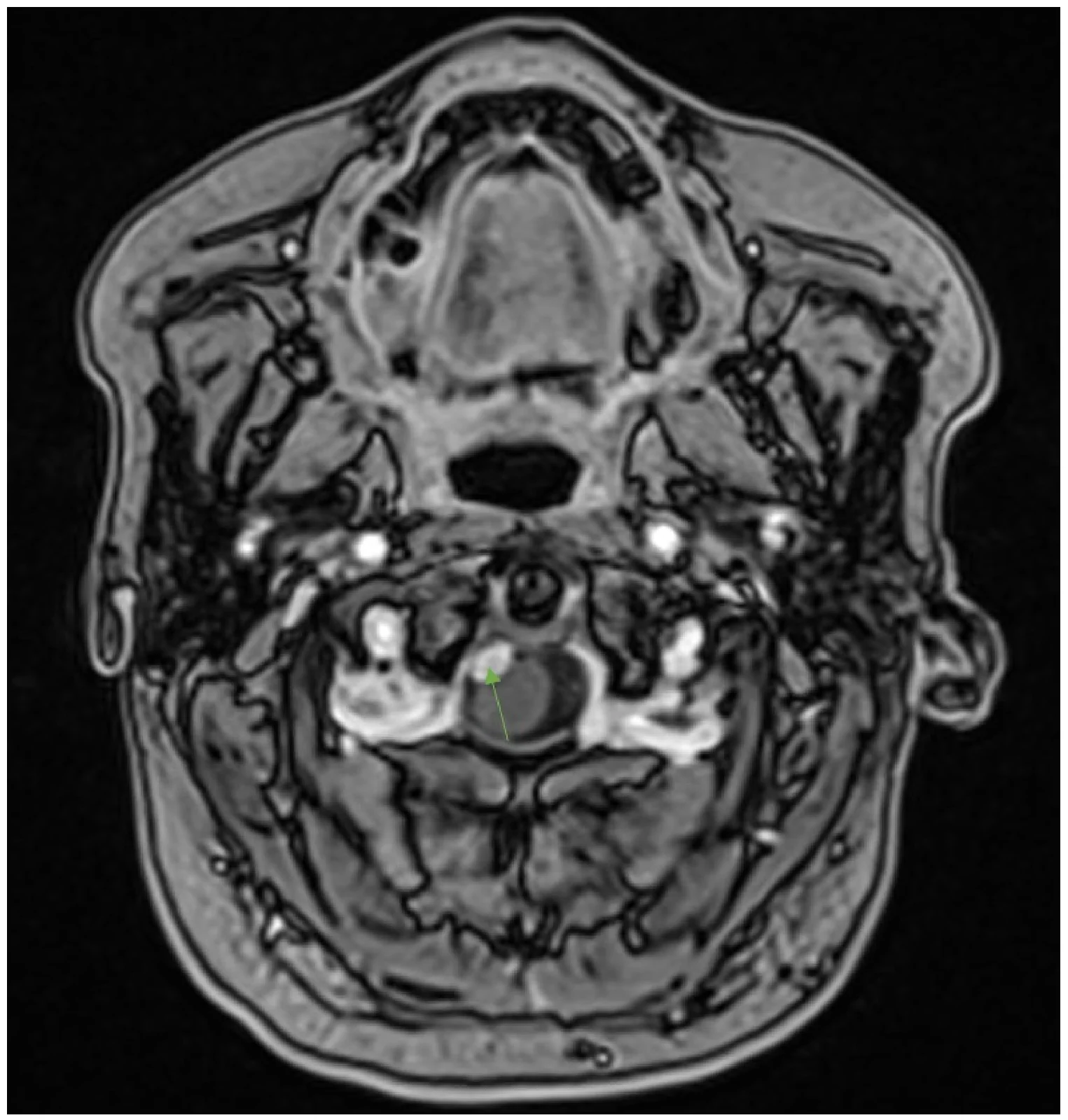
Preoperative post-gadolinium T1-weighted MRI demonstrating a second lesion on the ventral aspect of the spinal canal at the level of the odontoid process: axial plane (green arrow).

**Figure 3:**
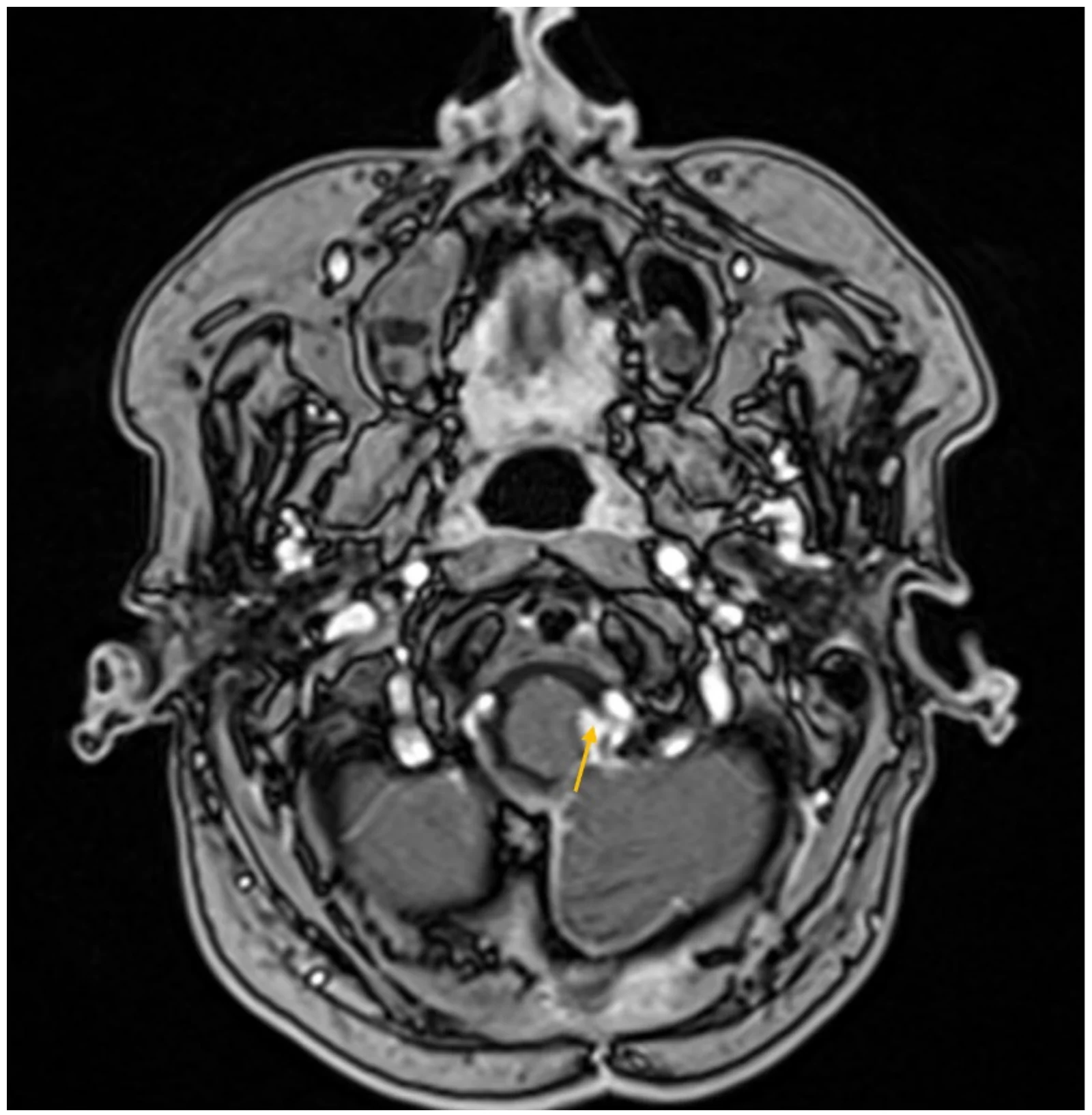
Postoperative post-gadolinium T1-weighted MRI demonstrating near-total resection with residual tumor adjacent to the vertebral artery–axial plane (yellow arrow).

**Figure 4:**
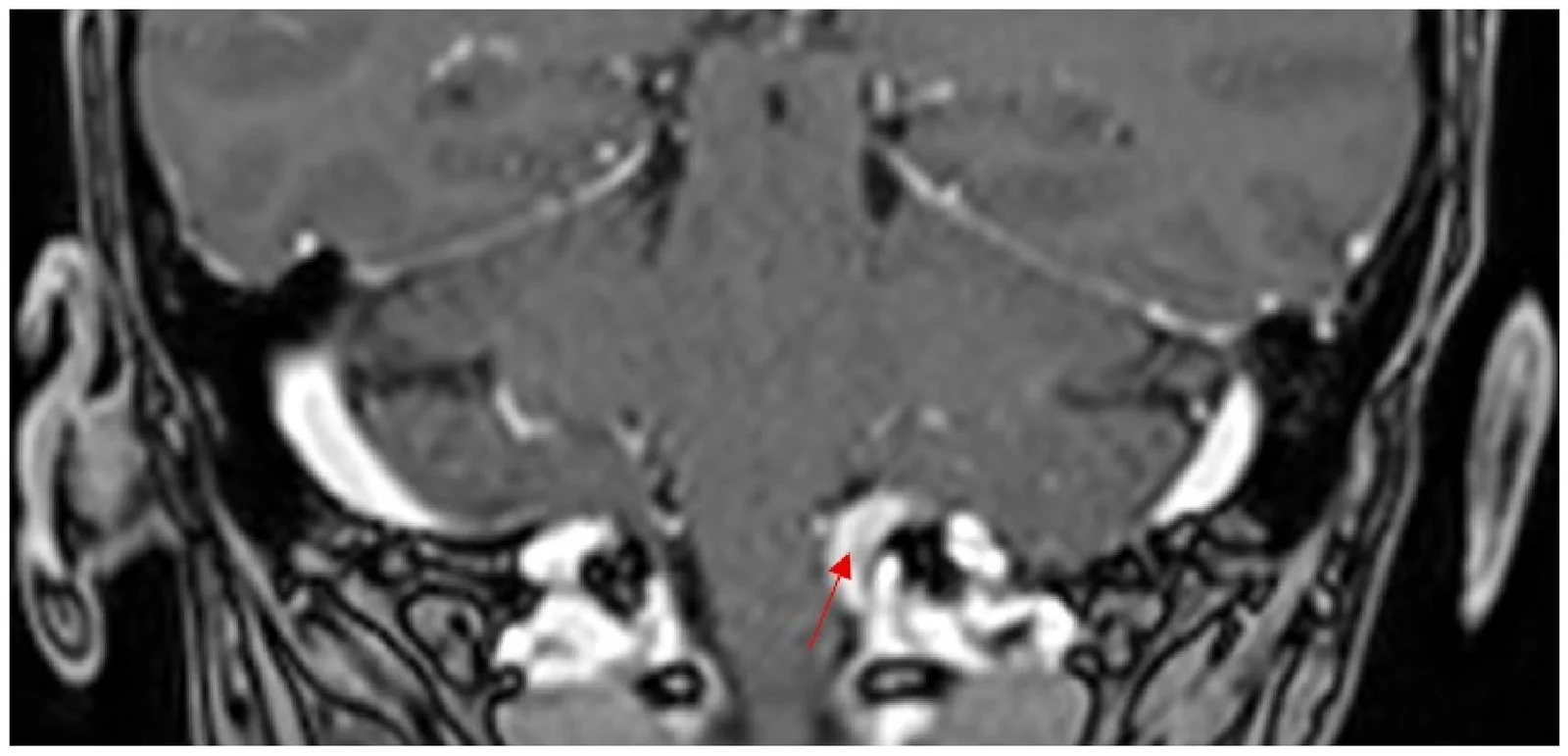
Postoperative post-gadolinium T1-weighted MRI after protonotherapy showing a stable residual tumor adjacent to the vertebral artery at the 6-month follow-up: coronal plane (red arrow).

## Data Availability

The datasets presented in this article are not readily available because because they constitute a clinical case report and contain patient-identifiable data. Requests to access the datasets should be directed to correspondence.

## Abbreviations

The following abbreviations are used in this manuscript:

CNS	central nervous system
CVJ	craniovertebral junction
GTR	gross total resection
STR	subtotal resection
